# Correspondence

**Published:** 1918-09

**Authors:** John P. Davin

**Affiliations:** 117 West 76th Street, New York


### CORRESPONDENCE

**This department is intended for the presentation of news and views not coming within the scope of other departments, and particularly to afford opportunity for discussion and criticism of views elsewhere expressed.**

117 West 76th Street, New York, Aug. 12, 1918.

Editor Buffalo Medical Journal:—

The new New York State Law to which you call attention in your August issue by which only licensed physicians may treat or prescribe for venereal diseases is in this respect a work of supererogation. It is another proof of the truth of which Ex. Chief Justice Cullen said of the statutes of this state, that "they are the greatest mass of legislative nonsense in existence." This law confers nothing on the physician which he did not already possess. To understand its true import the clause relating to the penalties inflicted upon druggists under its provisions for giving a copy of or renewing a prescription containing a remedy for venereal disease must be considered. In time this clause will be proved to be as unconstitutional as certain clauses of the Harrison law have just been proved to be. Meanwhile it advances the propaganda for the socialization of medicine as far as it relates to the monopoly by the state at the expense of the taxpayers of the treatment of venereal diseases. It is about time that the doctors woke up to the political activities of the state medical authorities at the expense of the profession.

JOHN P. DAVIN, M. D.

